# Does the Brain Detect 3G Mobile Phone Radiation Peaks? An Explorative In-Depth Analysis of an Experimental Study

**DOI:** 10.1371/journal.pone.0125390

**Published:** 2015-05-11

**Authors:** Suzanne Roggeveen, Jim van Os, Richel Lousberg

**Affiliations:** 1 Department of Psychiatry and Psychology, Maastricht University, Maastricht, Limburg, The Netherlands; 2 King’s College London, King’s Health Partners, Department of Psychosis Studies, Institute of Psychiatry, London, United Kingdom; Kagoshima University Graduate School of Medical and Dental Sciences, JAPAN

## Abstract

This study aimed to investigate whether third generation mobile phone radiation peaks result in event related potentials. Thirty-one healthy females participated. In this single-blind, cross-over design, a 15 minute mobile phone exposure was compared to two 15 minute sham phone conditions, one preceding and one following the exposure condition. Each participant was measured on two separate days, where mobile phone placement was varied between the ear and heart. EEG activity and radiofrequency radiation were recorded jointly. Epochs of 1200ms, starting 200ms before and lasting until 1000ms after the onset of a radiation peak, were extracted from the exposure condition. Control epochs were randomly selected from the two sham phone conditions. The main a-priori hypothesis to be tested concerned an increase of the area in the 240-500ms post-stimulus interval, in the exposure session with ear-placement. Using multilevel regression analyses the placement*exposure interaction effect was significant for the frontal and central cortical regions, indicating that only in the mobile phone exposure with ear-placement an enlarged cortical reactivity was found. Post-hoc analyses based on visual inspection of the ERPs showed a second significantly increased area between 500-1000ms post-stimulus for almost every EEG location measured. It was concluded that, when a dialing mobile phone is placed on the ear, its radiation, although unconsciously, is electrically detected by the brain. The question of whether or not this cortical reactivity results in a negative health outcome has to be answered in future longitudinal experiments.

## Introduction

Whether or not mobile phone radiation has an influence on human physiology and especially on brain activity is a research topic of increasing interest. Typically, people do not report bodily effects due to mobile phone use. Considered from a physical point of view, however, it is conceivable that the complex, sensitive electrochemical network that encompasses the brain, detects the electromagnetic radiation emitted by a mobile phone held against the head. An already known effect is that of heat transfer from mobile phones to the body [1–3], an effect which can be eliminated by the body. Next to this thermal process it is proposed that so called ‘non-thermal effects’ also take place while using a mobile phone. In short, it is thought that radio frequent electromagnetic fields (RF-EMF) may act as a trigger for the cellular stress response. No working mechanisms have been proven, but it is hypothesized that protein changes take place, which in turn could ultimately lead to undesired alterations like DNA damage which in turn could lead to tumor promoting effects and increase in permeability of the Sertoli cell barrier which could lead to male infertility [4–6]. In the mean while also studies are performed which focus on the measurement of direct electrophysiological effects of exposure to mobile phone radiation. Several studies with cortical activity as the dependent variable have been performed: experiments focusing on effects in waking and sleep EEG, as well as studies assessing event related potentials (ERP). In waking EEG studies, the most consistent finding is an increase in the alpha frequency band (8–12 Hz) activity during mobile phone radiation exposure [7–9]. In sleep EEG studies an increase of the sleep spindle frequency range (12–15 Hz) in non-rapid-eye-movement sleep has been reported repeatedly [10–13]. ERP is another often applied method to study brain activity, in which cortical stimulus-processing is investigated. Most studies in the field of mobile phone research investigate whether auditory stimuli (cochlear and brainstem auditory processes) are processed differently by the brain in the presence of a mobile phone [14]. The idea behind this hypothesized effect is that auditory organs absorb most of the radiation energy from the mobile phone in a dialing position [15]. However, not enough evidence has been reported to conclude that the presence of an active mobile phone alters the processing of these auditory stimuli [7,16]. In 2010, Carrubba and collegues proposed that mobile phone radiation pulses (instead of auditory stimuli), can be considered as stimuli [17]. Twenty participants were included and in 90% of the participants evoked potentials were observed at a latency of approximately 270 ms in response to mobile phone radiation pulses. Strictly speaking, this study investigated the ERP response of an unconscious/subliminal stimulus. Evidence has been reported that ERPs of subliminal stimuli have a comparable morphological structure to ERPs of supraliminal stimuli. However, the amplitudes produced by subliminal stimuli are smaller [18].

Recently a study was set up by our research team to investigate whether waking EEG frequency bands are influenced by mobile phone radiation [19]. In this study, significant radiation effects were found for the alpha, slowbeta, fastbeta, and gamma bands. Interestingly, it was found that the effects depended on placement location of the mobile phone (ear versus chest), the ear placement showing larger effects compared to the chest placement.

Considering a radiation pulse/peak as a stimulus, thereby following the idea proposed by Carrubba, is probably the most profound method to investigate whether radiation causes a direct change in the brain physiology. It was decided to re-analyse the above mentioned dataset of Roggeveen et al [17], to investigate whether a mobile phone radiation peak results in an event (radiation peak) related potential (ERP). Thus, instead of investigating change in the EEG frequency bands, in the present article the data of the same study was used to investigate the hypothesized presence of ERP in response to radiation peaks. In contrast to the study of Carrubba et al., the radiation source was a functioning mobile phone instead of a simulator and a 3G mobile phone network was used instead of a 2G network. As a consequence of the choice for a mobile phone as radiation source, a radiation detector was used to detect peaks (stimuli). Further, it was decided to apply a recently published technique to quantify ERP data: the so called ‘Event Related Fixed Interval Area’ (ERFIA) method. This method is appealing since it focuses on areas instead of specific peak amplitudes [20]. Because in some ERP studies, investigating subliminal stimuli, a P300 is observed, it was decided to create one ERFIA, ranging from 240–500ms, as the outcome measure of main interest. As a final methodological difference with Carrubba’s study, multilevel regression analysis was used, since EEG/ERP data contain a nested structure.

Since the results on Roggeveen’s dataset [19] demonstrated that the EEG-effects of mobile phone exposure were especially pronounced during mobile phone placement on the ear (compared to the chest), an a-priori placement*exposure interaction effect was expected.

## Materials and Methods

### Participants

Thirty-one female participants (mean age of 26.7; SD = 8.5), non-smoking, and without a medical history of cardiac or nervous system disorders were included. Four hours prior to the start of the session, no caffeine-containing beverages were used. No alcohol was used in the preceding 12 hours and sufficient night rest was ensured. After reading a document with detailed information about the study and having discussed any possible concerns with the researcher, subjects gave their verbal and written informed consent. Complete participation was compensated with €50,-.

### Experimental procedures

The study consisted of two sessions, each session taking place on a separate day, with a maximum of two days in between the two sessions. The experiments were conducted in an electrically non-shielded, room. The sequence of placement on the ear or heart was counterbalanced between the sessions. EEG was measured using shielded electrodes. Each shielded electrode had a separate ground plug, which was connected into a general ground-device. The following EEG electrodes were placed in accordance to the 10–20 system [21]: Fz, F3, F4, Cz, C3, C4, Pz, P3, P4, Oz, O1, and O2. All electrodes were fixed using conductive paste [22]. A reference was placed on each ear lobe. To check for possible eye movements, an electro-oculogram (EOG) electrode was placed 1 centimetre under the midline of both eyes. The electrodes were connected to a BrainAmp amplifier (Brain Products). Impedances were maintained below 5 kΩ. Spike artefacts due to radiation, which are mentioned in other articles [23,24], were not observed in the data. Both EEG and radiation data were sampled with 1000 Hz using Brain Vision Recorder software. Each participant was exposed to four consecutive 15 minute conditions during each session, according to the schedule shown in Table 1. There were three conditions with a sham phone, and one condition with a dialling mobile phone. The experimenter entered the room at the end of each 15 minute condition to change the phone. During this exchange of phones, no electrophysiological measurements took place. In the case of two consecutive sham phone conditions, the same procedure was followed (a second sham phone was placed). In order to ensure blinding, the order of the conditions was unknown to the participant, thus achieving a single-blind experiment. The experimenters were not blinded. In one session, the ‘dialling’ condition was in the second quarter of an hour and in the other session the ‘dialling’ condition was in the third quarter. This sequence order was balanced over the subjects. Subjects were not aware of the different phones used.

**Table 1:** Experimental design.

	15 minutes	15 minutes	15 minutes	15 minutes
**Day 1 or 2**	Pre-exposure (PRE)	Exposure (EXP)	Post-exposure (POST)	Not used
**Day 1 or 2**	Not used	Pre-exposure (PRE)	Exposure (EXP)	Post-exposure (POST)

The sequence was randomly determined in order to ensure blinding of the participant. In conditions labelled as ‘not used’, an identical sham phone was placed in the same way as in the pre- and post-exposure conditions.

### Exposure

- A 3G smartphone was used. During exposure conditions, the phone was dialled from a fixed line in another room. No sound was exchanged (mute settings), and vibration mode was off, in order to ensure that the participant could not identify the dialling condition.
- The SAR level of the phone was reported as 0.69 W/kg (head) in the manual.
- The sham phone was a non-functioning replica of the same weight and with the same characteristics as the functioning smartphone. In a pilot study before the start of the actual experiment, no evidence was found that participants could detect differences between the actual mobile phone and the sham phone.

Radiation activity was detected with a radiation detector (HF59B, Gigahertz Solutions), connected to an omnidirectional antenna. This detector was connected (from the DC output) to the BrainAmp headbox with an auxiliary plug. The detector was placed in the upright position, 30 cm above the table (at which the participant was sitting) and 20 cm left from the participant. In one of the two sessions, the phone/sham-phone was placed directly onto the left ear, ensuring that there was no contact between the phone and the EEG electrodes. The position of the phone was comparable to a typical dialling position, in an angle of approximately 45 degrees in relation to the perpendicular, tilted to the back of the head. During the other session, the phone was placed adjacent to the left side of the sternum, bordering the sternoclavicular joint. Previous tests showed that there was neither a direct interference of the mobile phone radiation on the shielded electrodes nor on the internal ADC converter of the amplifier. The rear side of the phone was placed on the skin in both sessions. The phone was fixed using an elastic band.

In order to investigate radiation exposure, a Network Analyzer, Agilent Technologies, E5061B ENA Series, 5 Hz—3 GHz was used. The frequency band operated in the following frequency: 1.9291 to 1.9397 GHz. A radiation peak as measured with the radiation detector, equalled a power of approximately 10 dbm measured next to the ear with the Network Analyzer.

In order to maintain the participant’s alertness and to guarantee a relatively stable mood, participants watched an affectively neutral documentary about the development of the earth. All experimental sessions were performed between 09.00 and 17.00 o’clock.

### Data reduction

EEG data was analysed offline with the software program BrainVision Analyser 2.0, Brain Products, München, Germany. EEG data was filtered using a high cut-off filter of 50 Hz and a low cut-off filter of 0.5 Hz, to remove noise from the data. In the exposure condition (EXP, 15 minute dialling exposure)mobile phone radiation peaks were identified and manually marked as such. Based on these markers time segments, further called epochs, of 1200 ms were created, starting 200ms before and lasting until 1000ms after the onset of a radiation peak. After segmentation a baseline correction (interval -200 to 0 ms) was performed. EOG data and radiation data were also divided into 1200 ms epochs. In the PRE and POST condition for each individual the same amount of markers was placed randomly. Twenty-millisecond ERFIAs [18] were calculated from -200 to 1000 ms, resulting in 60 ERFIAs per radiation peak per participant. For the analyses in this article, 4 aggregated ERFIAs were computed: -200 to 0ms, 0 to 240 ms, 240 to 500ms and 500–1000ms. On the average 40 radiation peaks were present during EXP. The same amount of epochs were randomly selected from PRE and POST. The three PRE-EXP-POST condition were reduced to one dichotomous variable, contrasting a dialing mobile phone (EXP) versus a sham phone condition (PRE and POST).

### Statistical analysis

Multilevel random regression analyses were used to investigate the effect of the radiation on the raw EEG outcome measures. The consecutive number of radiation peaks within each condition was the repeated measure variable. The multilevel regression analyses contained four levels: subject, session (every subject was measured on two separate days), condition (three conditions within each session) and radiation peak number (or its controls in PRE and POST). An autoregressive (AR1) covariance structure was used on the fourth level of radiation peak number in order to correct for the interdependency of the data. In all models a random intercept was included. An example of this model is shown in S1 Text.

The main outcome variable was computed as the area of 240–500 ms post stimulus. For the post-hoc analyses (see the results section) a second outcome variable was created: the area between 500 and 1000 ms post-stimulus. In order to demonstrate that there were no pre-stimulus effects a so called ‘baseline’ measure was computed, being the area from 200 ms pre-stimulus to zero.

To correct for eye blinking/movements, data with outlying electro-oculogram (EOG) activity were rejected from the analyses. Besides this rejection, the remaining EOG information was used as a covariate in the statistical models. In all regression models the outcome variable was predicted by the following main effects: exposure (dialling versus sham), placement of the phone (ear versus heart), EOG activity, session (day 1 or day 2) and radiation peak number. In addition, the interaction variable placement*exposure was included in all models.

P-values < = 0.05 were considered to be significant. All statistical analyses were performed with SPSS 22.0.

### Ethics statement

Approval was obtained from the medical ethics committee of the Academic Hospital Maastricht, on June, 6th, 2013.

## Results

**Fig 1:**
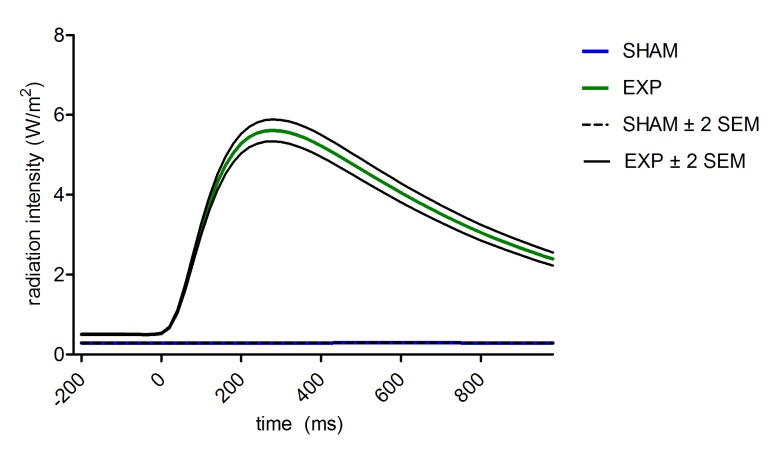
Mean radiation intensity of exposed and sham exposed epochs ± 2 standard error of the mean. The 4 grand averages of the non-conscious evoked related potentials of the twelve different locations are depicted in Fig 2. A visual inspection makes clear that the pre-stimulus baseline area (-200-0ms) does not show remarkable differences between the four grand averages. In the post-stimulus area, the exposed ear session is the only condition which has a distinct course. This effect is especially prominent in the 240–500ms frontal and central post-stimulus areas. Except Fz and F4, in which a P300-like peak can be detected, the morphology is not similar to that of most conscious ERP responses: a N200 is missing and the ERP has a smaller amplitude in general. Moving from frontal to occipital, the ‘P300 peak’ seems to diminish and a later effect, from 500–1000ms, becomes more noticeable. These are no obvious visual left-right hemispherical differences. Fig 3 shows the topography of the grand averages.

In order to visualize radiation intensity during the epochs, a graph was created with the mean radiation values in the two conditions (dialling/EXP versus sham) (Fig 1). As can be seen, the sham epochs (consisting of the PRE and POST conditions) show a flat line of radiation at approximately 0.1W/m^2^. This baseline-level can be considered as the background radiation level within the experimenting room. The radiation level during EXP epochs is, on the average, clearly above SHAM epochs. Even the radiation level of the -200ms to 0ms baseline period is significantly heightened. This is explained by a general raise in radiation level during phone calls. In EXP epochs an immediate increase of radiation from 0 ms onward can be observed. The maximum of 5.6 W/m^2^ is reached at 260 ms after the onset of the peak. There was variability between radiation epochs during the EXP (SDs ranged from MIN/MAX), however the difference between SHAM and EXP was significant (p<0.0001). Radiation mainly fluctuated between days, due to a different burden on the mobile phone network. It is worth noting, that after 1000 ms the radiation level has not yet returned to the level before the onset of the peak. The average amount of peaks during EXP per 15 minute session was 34.56 (2.30/minute) with a maximum of 100 peaks and a minimum of 14 peaks per 15 minutes. In total there were 2143 radiation peaks.

**Fig 2:**
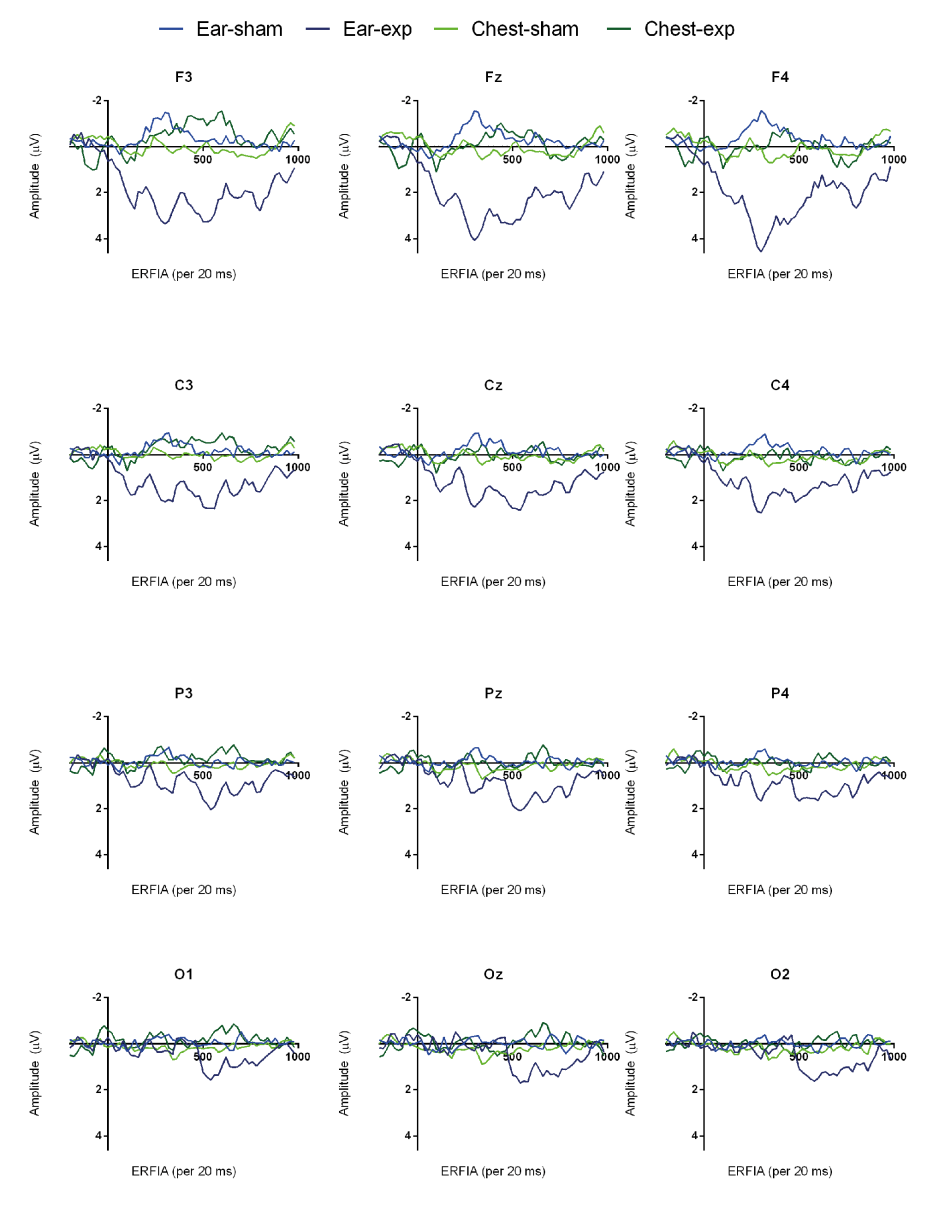
Event Related Fixed Interval Area (per 20ms) grand averages in twelve EEG locations.

**Fig 3:**
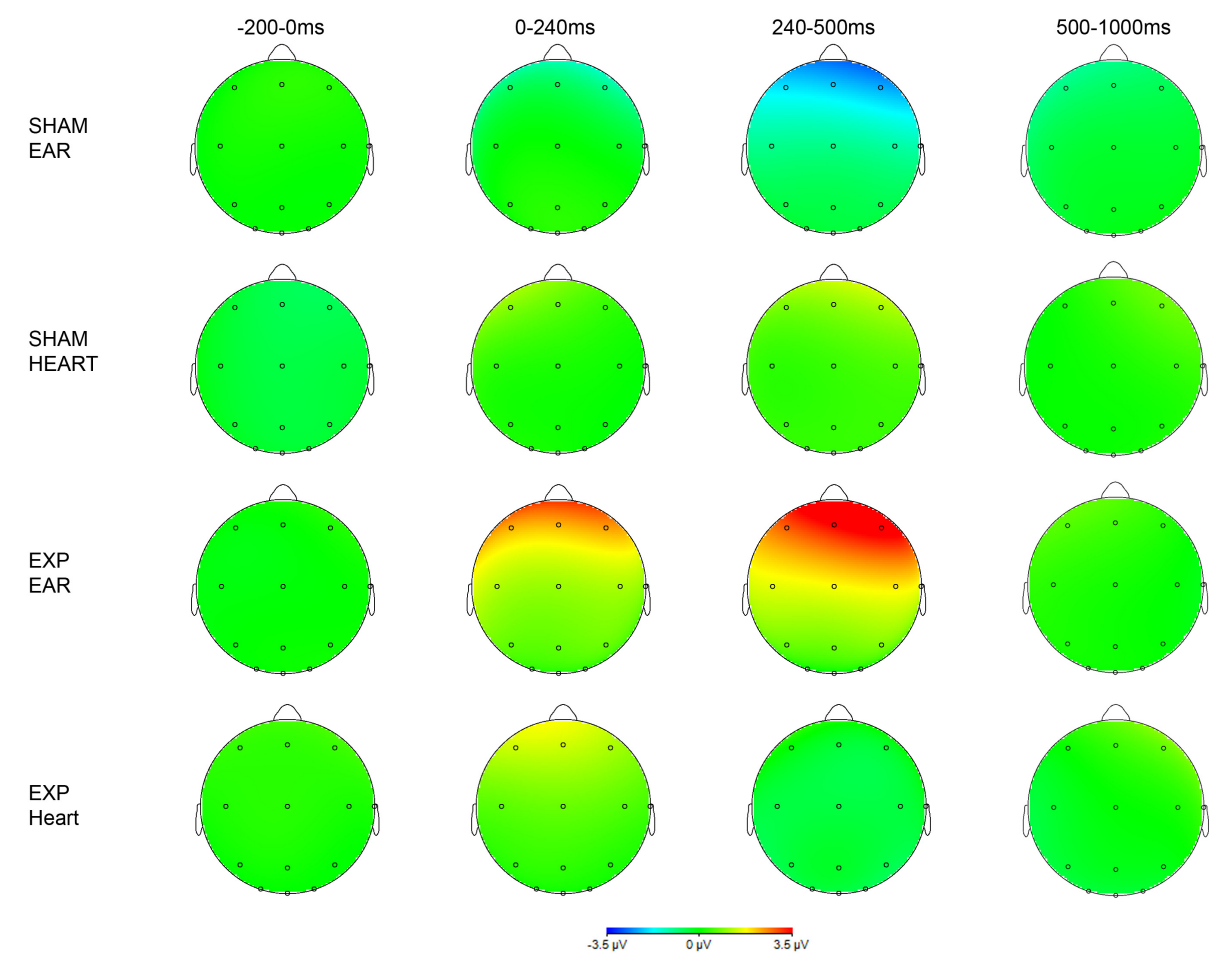
Topography of EEG activity. In Table 2 the t-values (and their corresponding p-values) of the main outcome variable are presented: the interaction effect between placement and exposure. As expected, the baseline (-200-0ms) never reaches significance. The same applies for the first post-stimulus period ranging from 0 to 240ms. In the critical range from 240–500ms the frontal and central regions show a significant elevation for the ear exposed condition compared to the other conditions. Based on the visual inspection of the grand averages, it was decided to perform a series of post hoc analyses on the ERFIA range of 500–1000ms post-stimulus. A significant elevation for the exposed ear condition was found for all electrodes except for F4 and C4. Finally, when correcting for multiple testing (12 locations) by means of the Bonferroni procedure (p_critical-corrected_ (0.05/12) = 0.004), an asterisk is placed in column ‘C’ when the p-value remains significant.

**Table 2:** T-values and p-values of the predictor; the interaction variable placement*exposure.

	-200 to 0ms			0 to 240ms			240 to 500ms			500 to 1000ms		
	t-value	p-value	C	t-value	p-value	C	t-value	p-value	C	t-value	p-value	C
**Fz**	1.889	0.059		-0.047	0.963		-3.030	0.002	*	-2.123	0.034	
**F3**	1.243	0.214		-0.543	0.588		-2.532	0.011		-2.648	0.008	
**F4**	1.106	0.269		-0.477	0.634		-3.121	0.002	*	-1.033	0.302	
**Cz**	1.880	0.060		0.163	0.871		-2.152	0.031		-2.436	0.015	
**C3**	1.379	0.168		-0.537	0.592		-2.567	0.010		-2.990	0.003	*
**C4**	1.360	0.174		-0.344	0.731		-2.442	0.015		-1.750	0.080	
**Pz**	.891	0.373		0.246	0.805		-0.721	0.471		-2.814	0.005	
**P3**	.841	0.400		-0.060	0.952		-1.360	0.174		-2.855	0.004	*
**P4**	.736	0.462		-0.328	0.743		-1.171	0.242		-2.379	0.017	
**Oz**	.540	0.589		0.372	0.710		0.583	0.560		-3.291	0.001	*
**O1**	.388	0.698		0.030	0.976		-0.008	0.994		-3.265	0.001	*
**O2**	.417	0.676		0.230	0.818		0.580	0.562		-2.956	0.003	*

The area in the first row is the dependent variable. An example of the statistical model used can be seen in S1 Text. C = significant after Bonferroni correction.

## Discussion

Several studies have demonstrated altered EEG frequency bands due to the presence of an active mobile phone (7–9). However, the causal mechanisms for this effect are, to our knowledge, not yet uncovered. Supposed electromagnetic radiation causes changes in EEG activity, a dose-response relationship should be demonstrable. In the present study it was hypothesized that 3G radiation peaks, short-term elevations of electromagnetic radiation, produce an immediate change in EEG activity, compared to a ‘non-radiation’ sham phone control condition. Radiation peaks were conceptualized as non-conscious stimuli which may produce event related potentials (ERPs). In other words, the key question to be answered was whether or not a 3G radiation peak (being a subliminal stimulus) can be detected by the brain, without assuming a response typical for a supraliminal stimulus. In the experimental design subjects were both exposed to a mobile phone placed on the ear, and to a mobile phone placed on the chest. These measurements took place on two separate days. The a-priori expected effect on the post-stimulus ERFIA range of 240–500ms, was evident: Not only a clear visual effect was observed, also the placement times exposure interaction was statistically significant, meaning an increase of cortical activity, only during the ear exposure condition and only in the frontal and central regions. Although this effect is not as large, regarding the amplitude, as observed in studies investigating supraliminal stimuli, it is comparable to effects of subliminal stimuli [18].

The non-significant interaction effects during the 200ms baseline may be considered as a validation of the just mentioned post-stimulus effects. Although the ERFIA grand averages of the exposed ear condition visually started from zero onwards, non-significant effects of the 0–240ms region were found. This might be due to a lack of power or may be the result of a non-optimal chosen ERFIA range.

By means of a visual inspection of the grand average ERFIA plots (Fig 2), it was noted that a second region (or to be considered as an extension of the first), from 500–1000ms, is affected during the ear-placement condition in all cortical regions. The placement times exposure interaction in this late region turned out to be significant in almost every EEG location. Although a profound interpretation of this late effect is lacking, it has to be noted that, in contrast to most ERP studies, the experimental stimulus (in this study a radiation peak) is active during the entire post-stimulus interval. Typically, the duration of a stimulus in an ERP study is relatively short (<100ms). The mean radiation peak (Fig 1), however, did not return to baseline after 1000ms. It therefore might be that EEG effects are more extended in time, compared to regular ERP studies. A post hoc explanation for the late (500–1000ms) effect might be that the maximum radiation intensity is reached after 260ms. Interestingly, this late effect turned out to be statistically greater compared to the 240–500ms effect.

To our knowledge, only the study of Carrubba et al [17] tested a comparable hypothesis to that of the present study. Although there are important methodological differences between these two studies (see introduction), it is worthwhile to compare the results. The timeframe in which an evoked potential was found in Carruba et al. was from 267 to 529ms post-stimulus. The largest ERFIA amplitudes of the present study were found in almost the same range. This resemblance might indicate a certain robustness of the radiation effect on cortical activity, quantified with ERP / ERFIA.

Several limitations of this study have to be mentioned. First, a relatively small and homogeneous population of 31 healthy females was included. A larger sample size, selected from the general population is needed to investigate the replicability and generalizability of the results. A second limitation concerns the, strictly speaking, non-experimental control of the radiation peaks. Although the exposure condition contained a lot of radiation peaks, there was no active control on both the size and the timing of these peaks. In addition, by using an active mobile phone, the brain is exposed to both electromagnetic and thermal processes. The usage of a device which experimentally simulates controlled radiation peaks comparable to that of a mobile phone might be preferred. On the other hand—and this was the main reason choosing a mobile phone in this study—a mobile phone provides the best representation of reality. Moreover, the finding of an immediate change in cortical activity after a radiation peak makes an explanation in terms of only thermal effects unlikely. Third, the design compared a 15 minutes dialling condition with a PRE and POST 15 minute sham phone condition. It could be argued that a more frequent alternating dialling schedule would be a more preferred method than the chosen ABA design. Fourth, since the duration of a mean radiation peak is longer than 1000ms, it would be interesting to experiment with a segment extending 1000ms. Fifth, the experimenter was not blinded. However, it is implausible that this lack of blinding has influenced the results. Finally, it seems unlikely that, executing the experiment in a non-shielded room (thereby allowing background radiation), has had a substantial effect on the EEG measured around radiation peaks.

In sum, this study demonstrates that non-consciously sensed radiation peaks, produced by a dialing 3G mobile phone, are detected by the brain in terms of short-term increased cortical activity. In addition, the ear placement specificity (compared to the chest) of the radiation effect on the cortex is striking. The crucial question whether or not the immediate effect of radiation on cortical activity may have an (negative) influence on health, cannot be answered yet. It would be ideal, but challenging, to perform longitudinal prospective research with differentially RF-EMF exposed groups in relation to several health outcomes. Next to EEG, transcranial magnetic stimulation might be used to test brain excitability, which has also shown changes in brain excitability due to mobile phone usage [25]. In addition, there is scope for investigation of effects at the cellular level, especially DNA change due to radiation, which will probably become more practical with the advent of novel imaging techniques.

## Supporting Information

S1 Datafile (SAV)

S1 Text Example of SPSS syntax for multilevel regression analysis. (DOCX)

S2 Text SPSS syntax. (SPS)
