# Supplementary material for: Does the Brain Detect 3G Mobile Phone Radiation Peaks? An Explorative In-Depth Analysis of an Experimental Study
Source: PLoS One. 2015 May 11;10(5):e0125390. doi: 10.1371/journal.pone.0125390 (PMC4427287; doi:10.1371/journal.pone.0125390)
Supplement: S1 Text — (DOCX) [file pone.0125390.s002.docx]

*MIXED Fz240_500 WITH EOGleft240_500 EOGright240_500 on_off ear_heart session peaknumber*

*/CRITERIA=CIN(95) MXITER(100) MXSTEP(10) SCORING(1) SINGULAR(0.000000000001) HCONVERGE(0,*

*ABSOLUTE) LCONVERGE(0, ABSOLUTE) PCONVERGE(0.000001, ABSOLUTE)*

*/FIXED= EOGleft240_500 EOGright240_500 on_off ear_heart session peaknumber on_off*ear_heart | SSTYPE(3)*

*/METHOD=REML*

*/PRINT=SOLUTION TESTCOV*

*/RANDOM=INTERCEPT peaknumber | SUBJECT(subject) COVTYPE(VC)*

*/REPEATED= peaknumber | SUBJECT(subject*session*condition) COVTYPE(AR1).*
